# Concurrent Tumor Treating Fields (TTFields) and Radiation Therapy for Newly Diagnosed Glioblastoma: A Prospective Safety and Feasibility Study

**DOI:** 10.3389/fonc.2020.00411

**Published:** 2020-04-21

**Authors:** Felix Bokstein, Deborah Blumenthal, Dror Limon, Carmit Ben Harosh, Zvi Ram, Rachel Grossman

**Affiliations:** ^1^Department of Neurosurgery, Tel Aviv Medical Center, Tel Aviv University, Tel Aviv, Israel; ^2^Neuro-Oncology Service, Tel Aviv Medical Center, Tel Aviv University, Tel Aviv, Israel; ^3^Institute of Radiotherapy, Tel Aviv Medical Center, Tel Aviv Medical Center, Tel Aviv University, Tel Aviv, Israel; ^4^Sackler Faculty of Medicine, Tel Aviv Medical Center, Tel Aviv University, Tel Aviv, Israel

**Keywords:** Tumor Treating Fields, radiotherapy, temozolomide, glioblastoma, safety

## Abstract

**Background:** TTFields are a loco-regional, anti-mitotic treatment comprising low-intensity alternating electric fields. In the EF-14 study of newly diagnosed glioblastoma (ndGBM), TTFields in combination with temozolomide (TMZ) significantly improved survival vs. TMZ alone. In preclinical studies TTFields had a radiosensitizing effect and increased the efficacy of radiation therapy (RT). This study prospectively evaluated the feasibility and safety of TTFields administered concurrently with RT and TMZ in ndGBM patients.

**Methods:** Patients with histologically confirmed ndGBM were treated with TTFields/RT/TMZ followed by adjuvant TMZ/TTFields. TTFields (200 kHz) were delivered for ≥18 hours/day with transducer arrays removed during RT delivery. RT was administered to the tumor bed in 30 fractions (total dose 60 Gy) combined with daily TMZ (75 mg/m^2^). In the adjuvant phase, patients received monthly TMZ (150–200 mg/m^2^ for 5 days) plus TTFields. Patients were followed for 24 months or until second disease progression. The primary outcome was safety of the combined therapies; secondary outcomes included progression-free survival (PFS) and overall survival (OS). Adverse events (AEs) were graded per CTCAE v4.0.

**Results:** Ten patients were enrolled at a single center between April and December 2017. Median age was 60.2 years, median Karnofsky Performance Score was 90.0, and 80% patients were male. Five (50%) patients had undergone tumor resection while the remainder had biopsy only. Eight patients experienced ≥1 RT treatment delay; delays were unrelated to TTFields treatment. All patients experienced ≥1 AE. Three patients suffered from serious AEs (urinary tract infection, confusional state, and decubitus ulcer) that were considered unrelated to TTFields. The most common AE was skin toxicity, reported in eight (80%) patients; all were of low severity (CTCAE grade 1–2) and were reported as related to TTFields treatment. Median PFS from enrollment was 8.9 months; median OS was not reached at the time of study closure.

**Conclusions:** Eighty percent of patients experienced grade 1–2 TTFields-related skin toxicity. No other TTFields-related toxicities were observed without an increase in RT- or TMZ-related toxicities as a result of combining TTFields with these therapies. Preliminary efficacy results are promising and warrant further investigation of concurrent TTFields/RT/TMZ treatment in ndGBM patients.

## Introduction

Tumor Treating Fields have been added in the last decade to the treatment arsenal of glioblastoma (GBM) patients. The EORTC/NCIC 22981/26981 study published in 2005 (1) established the previous standard of care for the treatment of newly diagnosed GBM. Following maximal safe surgical resection, patients who received daily temozolomide (TMZ) in combination with post-operative radiation therapy (RT), followed by adjuvant TMZ therapy, achieved a median overall survival (OS) of 14.6 months compared with an OS of 12.1 months in patients receiving RT alone (1). Despite this modest improvement in survival, GBM remains incurable, with only about 20% of patients surviving for 2 years (2), without TTFields. The recent phase III EF-14 multicenter, open-label, randomized trial compared the addition of TTFields to adjuvant TMZ treatment with TMZ alone in 695 patients with newly diagnosed GBM. The combination of TTFields with TMZ resulted in significant improvement in progression-free survival (PFS; 6.7 months vs. 4.0 months) and OS (20.9 months vs. 16.0 months) compared with TMZ alone (3, 4). The 5-year survival rate for patients in the TTFields plus TMZ group was 13% (vs. 5% for TMZ alone; *P* < 0.001), and the 2-year survival rate was 43% (vs. 31% for TMZ alone; *P* < 0.001) (4). Health-related quality of life was not compromised by the addition of TTFields (5).

TTFields are a unique treatment modality for GBM and other solid tumors, comprising low-intensity (~1–3 V/cm), intermediate frequency (100–300 kHz), alternating electric fields that act with specificity on rapidly dividing cancer cells (6–9). TTFields are US Food and Drug Administration (FDA)-approved at 200 kHz for newly diagnosed and recurrent GBM and at 150 kHz for malignant pleural mesothelioma. Multiple cytotoxic mechanisms are attributed to TTFields, including their effect upon microtubules and septin fibers of proliferating cancer cells, which disrupts mitosis and causes cell death, mitotic catastrophe, non-viable daughter cells, and cellular stress (6–11). TTFields also inhibit DNA damage repair (12), enhance replication stress (13), block cellular migration and invasion (14), and increase autophagy (15).

A synergistic effect between TTFields and RT was demonstrated in glioma cell culture, possibly through inhibition of double-stranded DNA damage repair mechanisms, increased mitotic catastrophe, and decreased glioma cell survival (16, 17). This preclinical evidence suggests that GBM patients may benefit from the concurrent administration of TTFields with RT and TMZ.

The objective of this prospective study was to evaluate the feasibility and safety of TTFields treatment administered concurrently with RT and TMZ in patients with newly diagnosed GBM.

## Methods

### Study Design

**Figure 1:**
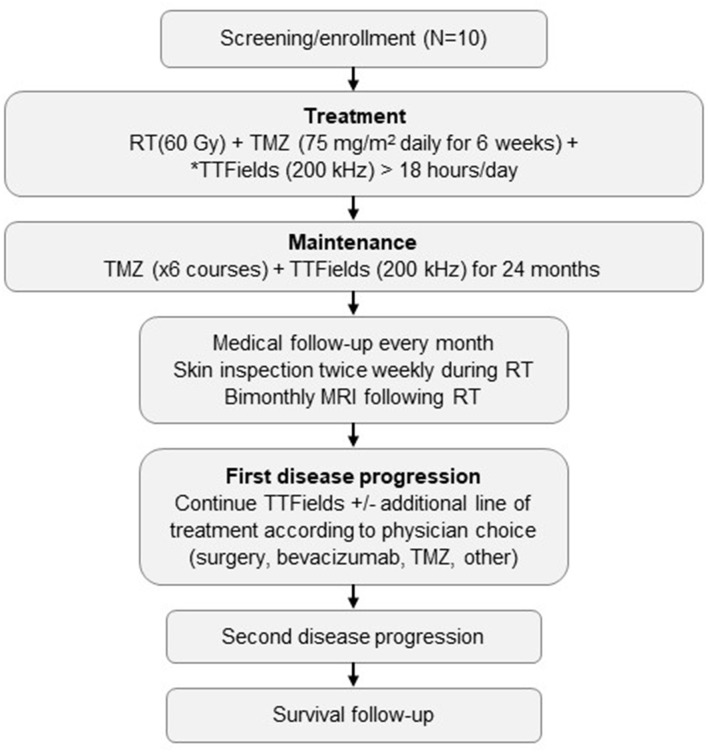
Study schema.*Starting ≥2 weeks before brain surgery and prior to or at the beginning of RT plus TMZ. TTFields are to be administered for >18 hours/day, with daily removal of transducer arrays during RT delivery. MRI, magnetic resonance imaging; RT, radiation therapy; TMZ, temozolomide; TTFields, Tumor Treating Fields.

This investigation was designed as a single-center, prospective, single-arm, open-label study with a planned enrollment of 10 patients (NCT03780569). All patients were required to provide written informed consent prior to registration in the study. Study was approved by the Ethics Committee of Tel Aviv Medical Center. The study design and treatment sequence are summarized in Figure 1.

### Study Outcomes

The primary outcome was the safety of combining TTFields with RT and TMZ as well as incidence of RT treatment delays during TTFields/RT/TMZ treatment. Secondary outcomes included median PFS and OS.

### Key Eligibility Criteria

Adult patients (≥18 years) with newly diagnosed and histologically confirmed supratentorial GBM after initial surgical resection or biopsy, with Karnofsky Performance Score (KPS) ≥70 and life expectancy ≥3 months, and who qualified as candidates for concurrent chemoradiotherapy were eligible. Pregnant women, patients with significant comorbidities at baseline prohibiting TMZ treatment, and patients with implanted devices in the brain were excluded.

### Treatments

Patients received standard low-dose TMZ (75 mg/m^2^/day for 6 weeks) together with focal RT at a total dose of 60 Gy given in 30 fractions. TTFields (200 kHz for >18 hours/day) were applied during the RT period. RT was planned for a single volume, composed of post-operative T1+ gadolinium changes (GTV) with a 1.5 cm margins and the surrounding T2 changes (CTV) and the addition of 3 mm margins for the planning target volume (PTV). All plans were delivered using Volumetric Modulated Arc Therapy (VMAT). After clinical and radiological evaluation of treatment response performed approximately 4 weeks after the end of TTFields/RT/TMZ, patients eligible for adjuvant treatment started monthly TMZ combined with TTFields treatment. Oral TMZ was administered at the conventional dosing regimen of 5 days of active treatment in a 28-day cycle. The first cycle was given at a dose of 150 mg/m^2^/day; starting from the second cycle, the TMZ dose was escalated to 200 mg/m^2^ in the absence of dose-limiting toxicity. TMZ treatment was administered until disease progression or for up to 6 months. TTFields treatment was given for up to 24 months and could be continued beyond tumor progression at the discretion of the investigator. The decision to discontinue TTFields due to unacceptable toxicity or tumor progression was based on the investigator's evaluation of the patient's clinical condition.

### Patient Assessment and Follow-Up

During RT, patients were assessed for safety which was evaluated according to the CTCAE (Common Terminology Criteria for Adverse Events) Version 4.0. The patients were seen twice weekly by a dermatologist and the transducer arrays were removed from the scalp to inspect the condition of the skin. Skin irritation or damage beneath the arrays was documented as an adverse event (AE) and graded according to CTCAE v4.0. The relationship to TTFields was assessed for each event based on the localization of skin irritation. Irritation outside of the areas covered by the transducer arrays was not considered to be related to TTFields treatment. Once dermatologic toxicities develop, interventions include, application of topical corticosteroids for contact dermatitis and topical antibiotics for infections at the time of array exchanges. After discontinuation of RT, monthly outpatient clinic visits were required until second disease progression or for up to 24 months, as long as the patients were receiving any protocol treatment and included medical examination and routine laboratory tests. TTFields usage time (patient compliance with treatment) was assessed monthly by electronic reports. Magnetic resonance imaging (MRI) was performed every 2 to 3 months following RT until second progression. Evaluation of treatment response was performed according to the MacDonald criteria.

## Results

Baseline patient and treatment characteristics are shown in Table 1. The median age was 60.2 years (range 42–72 years), the median KPS was 90 (range 80–100), and most patients were male (80%). Five (50%) patients underwent tumor resection and the remaining five had biopsy only.

**Table 1:** Patient baseline characteristics and treatments.

**Baseline characteristic**	**RT + TMZ + TTFields**
**Age (years)**	
Mean (SD)	59.9 (8.25)
Median (range)	60.2 (42–72)
**Karnofsky performance score**	
Median (range)	90.0 (80–100)
**Sex**, ***n*** **(%)**	
Male	8 (80.0)
**Extent of resection**, ***n*** **(%)**	
Biopsy	5 (50.0)
Tumor resection	5 (50.0)
**Number of TTFields cycles**	
Median (range)	9.0 (1–16)
**Follow-up duration**	
Median (range)	8.6 (2–15)
**Compliance in the RT period (%)**	
Mean (SD)	79.3 (8.36)
**Compliance in the first 3 months (%)**	
Mean (SD)	77.0 (10.56)

*RT, radiation therapy; SD, standard deviation; TMZ, temozolomide; TTFields, Tumor Treating Fields*.

Eight patients experienced at least one RT treatment delay: four patients had one delay, one patient had two delays, and three patients had three delays (Table 2). No delays in RT were related to TTFields treatment, in six patients the cause of the delays was holiday, and for two patients the causes were unknown.

**Table 2:** RT treatment delays.

**Number of RT delays**	**Number patients with RT delays (%)**	**Reasons for RT delays**
0	2 (20.0)	
1	4 (40.0)	Two unknown, two holiday
2	1 (10.0)	Holiday
3	3 (30.0)	Holiday

*RT, radiation therapy*.

AEs are shown in Table 3. All patients experienced at least one AE, and eight (80%) suffered from at least one systemic AE. Three (30%) patients had a serious AE (urinary tract infection, confusional state, and decubitus ulcer), and four (40%) patients had severe (CTCAE grade ≥3) AEs (lymphopenia, general physical health deterioration, intracranial hemorrhage plus neurological decompensation, and neurological decompensation), which were assessed as unrelated to TTFields treatment and were attributed to underlying disease, RT, or chemotherapy.

**Table 3:** AEs and severity.

	**RT** **+** **TTFields (*****N*** **=** **10)**		
**AEs**	**Total**	**Grade 1–2**	**Grade 3–4**
**Patients with** **≥** **1 AE**, ***n*** **(%)**	**10 (100)**	**6 (60)**	**4 (40)**
**Local complications**, ***n*** **(%)**	**8 (80)**	**8 (80)**	**0**
**Scalp skin complications**	**8 (80)**	**8 (80)**	**0**
Application site blisters	1 (10)	1 (10)	0
Application site erosions	3 (30)	3 (30)	0
Application site erythema	4 (40)	4 (40)	0
Contact dermatitis	4 (40)	4 (40)	0
Dermatitis	1 (10)	1 (10)	0
Eczema	1 (10)	1 (10)	0
Pruritus	1 (10)	1 (10)	0
Seborrheic keratosis	1 (10)	1 (10)	0
**Systemic complications**, ***n*** **(%)**	**8 (80)**	**4 (40)**	**4 (40)**
**Gastrointestinal disorders**	**3 (30)**	**3 (30)**	**0**
Abdominal pain	1 (10)	1 (10)	0
Constipation	2 (20)	2 (20)	0
Hematochezia	1 (10)	1 (10)	0
Nausea	1 (10)	1 (10)	0
Vomiting	1 (10)	1 (10)	0
**General disorders**	**6 (60)**	**5 (50)**	**1 (10)**
Asthenia	1 (10)	1 (10)	0
Chest pain	1 (10)	1 (10)	0
Fatigue	4 (40)	4 (40)	0
Gait disturbance	1 (10)	1 (10)	0
General physical health deterioration	1 (10)	0	1 (10)
Performance status decreased	1 (10)	1 (10)	0
Weight decreased	1 (10)	1 (10)	0
**Injury, poisoning, and procedural complications**	**1 (10)**	**1 (10)**	**0**
Spinal compression fracture	1 (10)	1 (10)	0
**Laboratory abnormalities**	**3 (30)**	**2 (20)**	**1 (10)**
Anemia	1 (10)	1 (10)	0
Blood creatine phosphokinase increased	1 (10)	1 (10)	0
Blood lactate dehydrogenase increased	2 (20)	2 (20)	0
Iron deficiency	1 (10)	1 (10)	0
Lymphopenia	1 (10)	0	1 (10)
Platelet count decreased	2 (20)	2 (20)	0
Vitamin D deficiency	1 (10)	1 (10)	0
**Nervous system disorders**	**5 (50)**	**3 (30)**	**2 (20)**
Cognitive disorder	1 (10)	1 (10)	0
Hemorrhage intracranial	1 (10)	0	1 (10)
Hemiparesis	1 (10)	1 (10)	0
Neurological decompensation	4 (40)	2 (20)	2 (20)
Quadrantanopia	1 (10)	1 (10)	0
Speech disorder	1 (10)	1 (10)	0
Other skin and subcutaneous tissue disorders	1 (10)	1 (10)	0
Decubitus ulcer	1 (10)	1 (10)	0
**Psychiatric disorders**	**2 (20)**	**2 (20)**	**0**
Abnormal behavior	1 (10)	1 (10)	0
Affective disorder	1 (10)	1 (10)	0
Confusional state	1 (10)	1 (10)	0
Delirium	1 (10)	1 (10)	0
Insomnia	2 (20)	2 (20)	0
Restlessness	1 (10)	1 (10)	0
**Renal and urinary disorders**	**2 (20)**	**2 (20)**	**0**
Urinary incontinence	1 (10)	1 (10)	0
Urinary tract infection	1 (10)	1 (10)	0

*AE, adverse event; RT, radiation therapy*.

**Figure 2:**
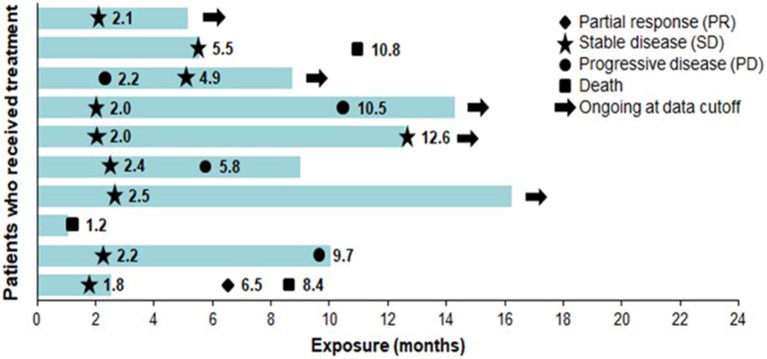
Exposure and response to TTFields + RT + TMZ and TTFields + TMZ maintenance therapy. Each bar represents one patient. Bar length represents study exposure time (months). Marker data labels represent event start since first TTFields dose date. RT, radiation therapy; TMZ, temozolomide; TTFields, Tumor Treating Fields.

**Figure 3:**
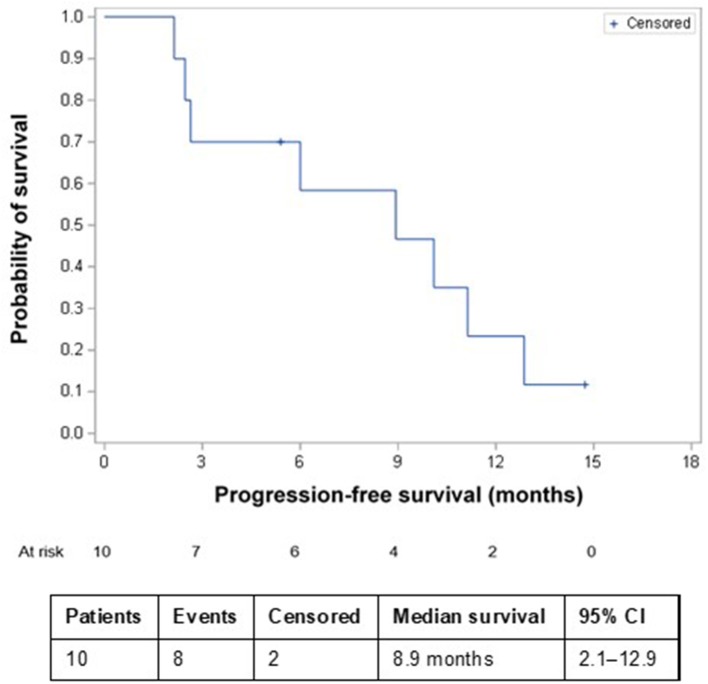
Kaplan–Meier progression-free survival in patients receiving TTFields + RT + TMZ and TTFields + TMZ maintenance therapy. CI, confidence interval; RT, radiation therapy; TMZ, temozolomide; TTFields, Tumor Treating Fields.

The most common AEs were dermatological (scalp skin) complications, reported in eight (80%) patients, and included application site erythema, erosions, blisters, dermatitis, seborrheic keratosis, eczema, and pruritus in the skin areas covered by the transducer arrays. All scalp skin complications were assessed to be related to TTFields and were of low severity (CTCAE grade 1–2). The skin reaction improved with use of topical corticosteroids. Regular relocation of the transducer arrays was necessary in order to allow for continuous treatment. Survival data are shown in Figure 2. The median follow-up time for the whole group was 8.6 (2–15) months and the average compliance rates in the radiation period and in the first 3 months after were 79 and 77%, respectively (Table 1). The median PFS was 8.9 months (95% CI: 2.1–12.9) (Figure 3), while the median OS was not reached. The PFS rates at 3 and 6 months were 70 and 58.3%, respectively. At the time of this report, seven patients are still alive, three of whom are without evidence of active tumor.

## Discussion

This pilot study, which assessed the feasibility and safety of combining TTFields treatment with initial RT and TMZ therapy in newly diagnosed GBM, demonstrated that the addition of TTFields to RT and chemotherapy was well-tolerated and did not exacerbate toxicities associated with either RT or TMZ treatment. Local dermatological (scalp skin) complications related to TTFields treatment were observed in 80% of patients, all of which were of low severity (CTCAE grade 1–2). Preliminary efficacy data from this study, with a median PFS of 8.9 months, are promising, particularly considering that half the recruited patients had only a biopsy before starting oncological treatment. In addition, this population included patients with early disease progression; these patients were excluded from the EF-14 study.

Preclinical studies demonstrated the rationale for combining TTFields with RT to treat GBM (16, 17). *In vitro* TTFields plus RT has synergistic antimitotic effects, which lead to increased mitotic catastrophe in GBM cells via inhibition of cell survival, regulation of cell cycle, and hindering of DNA repair (17). In glioma cell cultures, TTFields increase the kill rate of RT, thereby achieving greater RT efficacy by inhibiting or delaying DNA damage repair and promoting cell death (16). When the initiation of TTFields was delayed until the end of RT treatment, the overall treatment efficacy diminished. This suggests that TTFields radiosensitized cancer cells, which may provide a therapeutic advantage in treating RT-resistant glioma cells (16). Exposing cells to TTFields immediately following ionizing radiation resulted in increased chromatid aberrations and a reduced capacity to repair DNA double-strand breaks, suggesting that TTFields induce a state of “BRCAness,” leading to enhanced sensitivity to ionizing radiation, which underscored a rationale for the use of TTFields combined with RT (12).

Median PFS in the landmark EORTC/NCIC study was 6.9 months in the RT plus TMZ treatment group and 5.0 months in the TMZ alone group, with patients starting treatment a median of 5 weeks after diagnosis (1). In that study, just 17% (48 patients) had only a biopsy before initiating RT plus TMZ treatment, while the remaining 83% (239 patients) underwent complete or partial resection. In the EF-14, the median PFS from randomization was 6.7 months in the TTFields-temozolomide group and 4.0 months in the temozolomide-alone group (HR, 0.63; 95% CI, 0.52–0.76; *P* < 0.001) (4). The PFS result from our study (median of 8.9 months) is encouraging compared with the PFS of 6.9 months and 6.7 months in the EORTC/NCIC and the EF-14 studies. Although patients in our study continued on TTFields after RT, it is notable that 50% had only a biopsy before initiating TTFields concurrent with RT and TMZ. Two separate pilot studies have recently demonstrated the safety and feasibility of concurrent RT and TTFields therapy utilizing scalp-preserving chemoradiation without removing the TTFields transducer arrays (18, 19).

Concurrent chemotherapeutic regimens added to the EORTC protocol represent a potential therapeutic strategy—notably the combination of the anti-angiogenesis agent bevacizumab (BEV) with TMZ is one such strategy. A meta-analysis compiling 6 randomized controlled trials combining BEV with TMZ for ndGBM indicated a 33% reduction in the risk of disease progression with no effect on OS (20). Anti-angiogenic therapies such as BEV may further increase the risk of skin-related AEs associated with TTFields (21). Concurrent therapies for ndGBM with TTFields that may exacerbate skin irritation underscore the necessity of effective prophylactic strategies to minimize the occurrence of skin toxicities associated TTFields (e.g., proper shaving techniques, scalp cleansing, and array relocation) as well as AE specific treatment-based strategies (e.g., topical or oral antibiotics, isolation of affected skin, or topical corticosteroids) to manage skin irritation (22). In this study the mild to moderate skin reactions associated with TTFields where managed with corticosteroids and relocation of the transducer arrays allowing participants to continue TTFields therapy uninterrupted.

Limitations of this study are that it is a single-center study with a small sample size. However, the positive results, as well as the preclinical evidence, are hypothesis-generating and justify further clinical evaluation of the proposed TTFields treatment regimen.

In summary, combining TTFields with RT and TMZ following resection was safe and well-tolerated in newly diagnosed GBM patients. Based on results of this pilot study, a phase II study (NCT03869242) enrolling 60 newly diagnosed GBM patients testing this regimen is underway.

## Data Availability Statement

The raw data supporting the conclusions of this article will be made available by the authors, without undue reservation.

## Ethics Statement

The studies involving human participants were reviewed and approved by Ethics Committe, Tel Aviv Medical Center. The patients/participants provided their written informed consent to participate in this study.

## Author Contributions

All authors contributed to the study concept and drafting, and review of manuscript drafts.

## Conflict of Interest

RG received travel support from Novocure. ZR received grant support and owns Novocure stock options. The remaining authors declare that the research was conducted in the absence of any commercial or financial relationships that could be construed as a potential conflict of interest.
